# Combining the use of a fetal fraction‐based risk algorithm and probability of an informative redraw in noninvasive prenatal testing for fetal aneuploidy

**DOI:** 10.1002/jgc4.1208

**Published:** 2019-12-23

**Authors:** Peter Benn, Kimberly Martin, Trudy McKanna, Elizabeth Valenti, Paul Billings, Zachary Demko

**Affiliations:** ^1^ UConn Health Farmington CT USA; ^2^ Natera, Inc. San Carlos CA USA

**Keywords:** cell‐free DNA, fetal fraction, genetic counseling, genetic testing, noninvasive prenatal testing, prenatal diagnosis, prenatal screening, trisomy

## Abstract

Some women undergoing noninvasive prenatal testing (NIPT) do not receive an informative result due to low fetal fraction (FF). A proportion of these are at increased risk for fetal trisomy 13, 18, or triploidy, while others have no change from their prior risk. Women with an initial uninformative NIPT need to be counseled about any such change in their risk for fetal abnormality and also the probability that a redraw will be informative. To help in the decision making, we reviewed a dataset of single nucleotide polymorphism‐based NIPT with uninformative results where a redraw was received. Risk for trisomy 13, 18, or triploidy was evaluated using a fetal fraction‐based risk (FFBR) algorithm. Risk‐unchanged women were further analyzed using a regression model to determine the likelihood of an informative redraw. Of 2,644 women with an uninformative NIPT and a redraw, 1,147 (43.4%) were high risk for trisomy 13, 18, or triploidy. 1,497 (56.6%) were risk unchanged and, of these, 975 (65.1%) cases had an informative redraw (i.e., risks were available for 2,122 (80%) of those initially classified as uninformative). The regression model for the risk‐unchanged cases provided a new table for predicting an informative redraw. Likelihood of a successful redraw was significantly (*p* < .001) dependent on the initial FF, maternal weight, and time between blood draws. We conclude that the FFBR algorithm and the predictive model for an informative redraw provide complementary additions in the management of women presented with an initially uninformative SNP‐based NIPT due to low FF. We suggest approaches for the counseling and follow‐up testing for women with an initially uninformative NIPT.

## 1 INTRODUCTION

Noninvasive prenatal testing (NIPT) for fetal chromosome abnormalities, based on the analysis of cell‐free DNA (cf‐DNA) in maternal plasma, is now widely available as a clinical service (Bianchi & Chiu, 2018; Cuckle, Benn, & Pergament, 2015). Test performance is related to the proportion of placentally derived DNA, relative to the amount of maternal DNA (Wright, Wright, & Nicolaides, 2015). This proportion, referred to as the fetal fraction (FF), is measured using different methodologies by the various laboratories offering this testing (Wataganara, Bui, Choy, & Leung, 2016). FF is known to be inversely proportional to maternal weight and weakly dependent on gestational age during the time that most NIPT is carried out (Dar et al., 2014; Wang et al., 2013). Trisomy 18, trisomy 13, digynic triploidy, and pregnancy loss are associated with low FF (McKanna et al., 2019; Revello, Sarno, Ispas, Akolekar, & Nicolaides, 2016).

We recently reported that FF could be used as a biomarker to identify the sub‐group of women who do not receive a NIPT result using a SNP‐based NIPT but are at significantly increased risk for trisomy 18, trisomy 13, triploidy, and pregnancy loss (McKanna et al., 2019). Using a risk cutoff of 1 in 100 in this fetal fraction‐based risk (FFBR) algorithm, 22% of the high‐risk group had abnormal pregnancy outcomes. This algorithm has been introduced into clinical practice, and instead of receiving an uninformative NIPT result, increased risk status for the relevant conditions is reported. These women can therefore be promptly offered further counseling, ultrasound, and the option of diagnostic testing. Conversely, those women with FFBR <1 in 100 showed no increased risk for any of these conditions and submission of a repeat sample could be a reasonable option.

When considering the options for further evaluation after an initial ‘no result’ from NIPT, it is helpful to consider the likelihood that a repeat sample would yield a high confidence result. We have examined the reasons for the inability to obtain an initial result using SNP‐based NIPT and developed a mathematical model for predicting the probability that a result would be obtained from a redraw (Benn, Valenti, Shah, Martin, & Demko, 2018). However, that analysis included women who would have a high risk for trisomy 18, 13, and triploidy using the FFBR algorithm. For these high‐risk women, proceeding directly to ultrasound and possibly an invasive test, rather than a re‐sampling, would be most appropriate.

The application of the FFBR algorithm therefore prompted a need to reformulate the informative redraw predictive model with a focus only on those women with no increased for trisomy 13, trisomy 18, or triploidy (the FFBR ‘risk‐unchanged’ sub‐group). In this paper, we provide the revised predictive formula. We also discuss the complex options and pathways for the clinical management of women with an initial no result with the goal of facilitating counseling.

## 2 MATERIALS AND METHODS

### 2.1 Participants

We reviewed 159,574 SNP‐based NIPT samples received by Natera, Inc. between January 1, 2016, to October 1, 2016, to identify all cases where there was an initial ‘No‐Result’ and a redraw was carried out. A full audit of these cases has been presented elsewhere (Benn et al., 2018). This dataset included 2,959 cases where a result was not provided on the initial sample due to low FF or low confidence data and the following criteria were met for the inability: a repeat sample was received, the difference in the estimated date of delivery provided for the two draws was within 7 days, and the redraw was received within 28 days of the first sample.

### 2.2 Data analysis

For each initial sample, a FF z‐score (the number standard deviations that the FF departed from the mean after adjustment for the patient's weight and gestational age) and the FFBR score were calculated, as described elsewhere (McKanna et al., 2019). FFBR could be calculated for 2,644 cases in the total cohort of 2,959; there were 315 cases where missing maternal weight data precluded the calculation of risk (Figure S1). A risk assignment of ≥ 1% was used to define the high FFBR group.

Comparison of the rate of suspected chromosome abnormalities in cases with a high FFBR result vs. the rate of suspected chromosome abnormalities in the FFBR risk‐unchanged group was carried out using Fisher's exact or chi‐square tests. To calculate the net proportion of cases with a result of any type, we considered cases with a high FFBR score as informative and those cases with a result obtained from a follow‐up plasma sample as informative.

For the purposes of counseling women on the chance that a redrawn sample would return a result for risk‐unchanged women, a binary logistic regression analysis was carried out for the FFBR risk‐unchanged sub‐group. This predictive modeling was carried out using SPSS (IBM). A *p*‐value of < .05 was considered significant.

## 3 RESULTS

Table 1 summarizes the number of pregnancies, demographic characteristics, FF details, days between draws, and successful redraws for the ‘no result’ cases included in this study. As expected, women with high FFBR were older and had a lower maternal weight and lower FF. This reflects the contribution of these variables in the FFBR algorithm. Overall, 1,497/2,644 (56.6%) women had an unchanged risk for fetal chromosome abnormality by the FFBR algorithm. Of these 1,497 cases with risk unchanged, 975 (65.1%) had an informative redraw (Figure S1). Of the 2,644 cases initially classified as uninformative, 2,122 (80%) were considered high FFBR or had risk information after redraw.

**Table 1:** Number of pregnancies, mean and range for demographics, fetal fraction, change in fetal fraction and times between blood draws, and redraw success rates for cases with high and unchanged risk by the FFBR algorithm

	All cases	High FFBR	Risk unchanged
Number of pregnancies	2,644	1,147	1,497
Maternal age (years)	32.7 (15–48)	36.1 (16–48)	30.1 (15–43)
Gestational age at first draw (weeks)	12.2 (9–29.1)	11.8 (9–22.7)	12.5 (9–29.1)
Maternal weight (lbs.)	206 (90–558)	176 (90–440)	229 (98–558)
Initial fetal fraction (%)	3.1 (0.9–8.7)	2.7 (0.9–6.7)	3.4 (0.9–8.7)
Change in fetal fraction (%)	1.3 (−3.0–21.2)	0 (−2.6–21.2)	1.0 (−3.0–15.0)
Days between draws	14.2 (5–28)	13.6 (5–28)	14.7 (5–28)
Redraw informative	1,673 (63.3%)	698 (60.9%)	975 (65.1%)

Abbreviation: FFBR; fetal fraction‐based risk.

**Figure 1:**
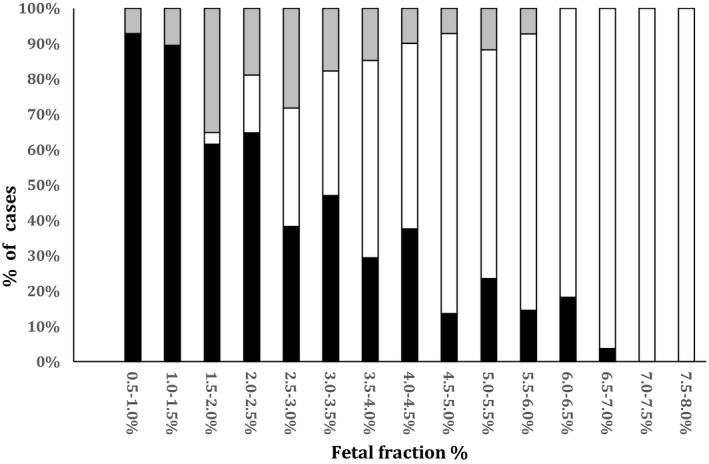
Proportions of informative cases based on fetal fraction‐based risk (FFBR), or redraw for various fetal fractions. Black boxes denote the proportion of cases determined to be high risk as a result of the FFBR algorithm (FFBR high risk); white boxes denote the proportion of cases that are provided a risk as result of an informative redraw; gray denotes cases that are FFBR risk unchanged, receive a redraw and the testing remains uninformative. If all women with risk unchanged by the FFBR algorithm undergo a redraw, the expected proportion of uninformative results would be 0.7% (weighted average of the gray boxes) See text

In the full cohort, a redraw was recommended due to low FF or low confidence data in 3.55% of all cases (8,605 of 242,607) (Benn et al., 2018). If each of these women had received a FFBR evaluation, and those with risk unchanged received a redraw, the net percentage of cases with an uninformative redraw after two attempts would be 3.55% × 56.6% × (100%–65.1%) = 0.7%. Figure 1 illustrates the relative distribution of cases that are either high FFBR, provided a result following a redraw, or no result after redraw.

Table 2 indicates the proportion of women who would be considered FFBR high risk based on the FFBR algorithm for various initial sample FFs. For women with FF <1.5%, 90% would be considered high risk based on their FFBR calculation. However, a high FFBR was not confined to those with the lowest FF. For women with FF >6%, 10% were considered high risk. For all women with FF <4% (a commonly used lower level threshold that has been used for acceptable testing in NIPT), 48% would be considered FFBR high risk.

**Table 2:** Proportion of cases that are FFBR high risk for different observed levels of fetal fraction at the first draw

Fetal fraction (%)	High FFBR	Risk unchanged	Total	% high FFBR
<1.5	64	7	71	90.1
1.5–2.0	112	70	182	61.5
2.0–2.5	295	161	456	64.7
2.5–3.0	289	466	755	38.3
3.0–3.5	191	215	406	47.0
3.5–4.0	78	187	265	29.4
4.0–4.5	68	113	181	37.6
4.5–5.0	21	133	154	13.6
5.0–5.5	16	52	68	23.5
5.5–6.0	8	47	55	14.5
>6.0	5	46	51	9.8
All	1,147	1,497	2,644	43.4

Abbreviation: FFBR; fetal fraction‐based risk.

Within the 975 cases that had risk unchanged and had an informative SNP‐based result on redraw, there were 20 cases (2.1%) that were reported as high risk or suspected as having a fetal chromosome abnormality (Table 3). The 20 cases included 10 trisomy 21, 1 trisomy 18, 2 trisomy 13, and seven sex chromosome abnormalities. No samples were interpreted as either multiple gestations or triploidy. For the 698 cases that had a high FFBR and a SNP‐based result on redraw, 34 (4.9%) had a high risk for aneuploidy; 16 trisomy 21, 6 trisomy 18, 5 trisomy 13, three sex chromosome abnormalities, and four multiple gestation/triploidy. The difference in these rates for these two groups was statistically significant (*p* < .001) but was largely attributable to trisomy 13, trisomy 18 and multiple gestations/triploidy (*p* < .001) and not trisomy 21, and sex chromosome abnormalities (*p* = .17). The excess in these results was therefore consistent with the FFBR model expectations (McKanna et al., 2019). Actual pregnancy outcomes were not collected for this cohort.

**Table 3:** NIPT SNP‐based high‐risk results for cases that had an informative redraw

NIPT result	High FFBR (%)	Risk unchanged (%)	Significance*
Results associated with high FFBR			
Trisomy 13	5 (0.7)	2 (0.2)	
Trisomy 18	6 (0.9)	1 (0.1)	
Possible twin/triploidy	4 (0.6)	0 (0.0)	
Subtotal	15 (2.1)	3 (0.3)	*p* < .001
Results not associated with high FFBR			
Trisomy 21	16 (2.3)	10 (1.0)	
Sex chromosome abnormality	3 (0.4)	7 (0.7)	
Subtotal	19 (2.7)	17 (1.7)	*p* = .17
All high‐risk calls	34 (4.9)	20 (2.1)	*p* = .0013
Low‐risk result	664 (95.1)	955 (97.9)	*p* = .0013
Total	698	975	

Abbreviation: FFBR; fetal fraction‐based risk.

* Comparison of the positive call rate in the high FFBR group vs. the risk‐unchanged group. Fisher exact or Pearson chi‐square tests.

Table S1 summarizes the statistical parameters derived for the revised predictive model for an informative redraw for FFBR risk‐unchanged women; that is, those for whom a repeat sample could be reasonable option. Initial FF, maternal weight, and time between draws were all statistically significant in the model (*p* < .001). Table 4 provides modeled probabilities for an informative redraw for women 75–300 lbs (34–136 kg) and fetal fractions 1.5%–6% with the redraw carried out at 8 days after the initial draw (previously established as the earliest and most practical time) (Benn et al., 2018).

**Table 4:** Expected informative redraw rates (%) at 8 days for women with unchanged risk by the FFBR algorithm

Maternal weight lbs (kg)	Fetal fraction (%) from first blood draw									
	1.5	2.0	2.5	3.0	3.5	4.0	4.5	5.0	5.5	6.0
75 (34.0)	35	46	58	68	77	84	89	93	95	97
100 (45.4)	33	43	54	65	75	82	88	92	95	97
125 (56.7)	30	40	51	62	72	81	87	91	94	96
150 (68.0)	27	37	48	59	70	78	85	90	93	96
175 (79.4)	25	34	45	56	67	76	84	89	93	95
200 (90.7)	23	32	42	53	64	74	82	88	92	95
225 (102.1)	21	29	39	50	61	71	80	86	91	94
250 (113.4)	19	26	36	47	58	69	78	85	90	93
275 (124.7)	17	24	33	44	55	66	75	83	88	92
300 (136.1)	15	22	31	41	52	63	73	81	87	91

Abbreviation: FFB; fetal fraction‐based risk.

## 4 DISCUSSION

Recent studies have shown that there is a subset of women with a ‘no result’ on NIPT that are at increased risk for trisomy 18, trisomy 13, digynic triploidy, and pregnancy loss but not trisomy 21 or monosomy X (McKanna et al., 2019). This group of cases can be distinguished using the FFBR algorithm that utilizes FF as a biomarker after adjustment for maternal weight, maternal age, and gestational age. We show that using the FFBR algorithm materially reduces the proportion of women with entirely uninformative results. Our analysis is comprised of a subset of approximately 3.6% of all NIPT cases where an initial result was uninformative due to low FF or low confidence and a repeat specimen was submitted. Under a revised protocol in which the FFBR algorithm was applied and redraw was carried out only for those women with risk unchanged, the number of women with an uninformative NIPT could be reduced to as little as 0.7% of the full cohort.

### 4.1 Prior studies

For women where a result was not provided from an initial sample, whose risks were unchanged by the FFBR algorithm, and had an informative redraw, 2.1% had a high‐risk call from the second draw. This rate is similar to the rate (1.8%) previously reported for all women referred for NIPT (Dar et al. (2014)). This observation provides additional evidence that this group of women can be counseled that their uninformative result does not measurably alter their prior age‐related risk (McKanna et al., 2019). Conversely, the group of women with high FFBR had a 4.9% rate of high‐risk results upon redraw with the excess risk attributable to trisomy 13, trisomy 18, and triploidy. In our previous study, we reported a 7.1% rate of chromosome abnormality in high FFBR women with additional abnormal karyotypes likely among the 14.7% of cases that resulted in pregnancy loss (McKanna et al., 2019). The earlier study was based on follow‐up studies of all women with high FFBR and included women with uninformative repeat testing where FF was especially low. Very low FF is especially strongly associated with triploidy.

### 4.2 Practice implications

Information on maternal age, weight, and gestational age should be provided with NIPT test submissions, and this information is needed for those cases requiring FFBR and informative redraw assessments. For FFBR algorithm risk‐unchanged women, prediction of the chance of an informative redraw should be helpful in choosing between the various available prenatal screening and diagnostic options. We provide a modified set of statistical parameters for calculating the chance of a successful redraw when there is an initial no result (Table S1 and Table 4). If a single redraw, performed a minimum of 8 days after the first sample, still does not provide an informative result, we do not advocate obtaining a third NIPT sample. Similar to the problem with repeat testing in maternal serum screening, regression to the mean can potentially result in falsely reassuring results (Haddow, Palomaki, Wald, & Cuckle, 1986). We also do not recommend a third NIPT attempt due to advancing gestational age and the need for timely completion of all testing.

**Figure 2:**
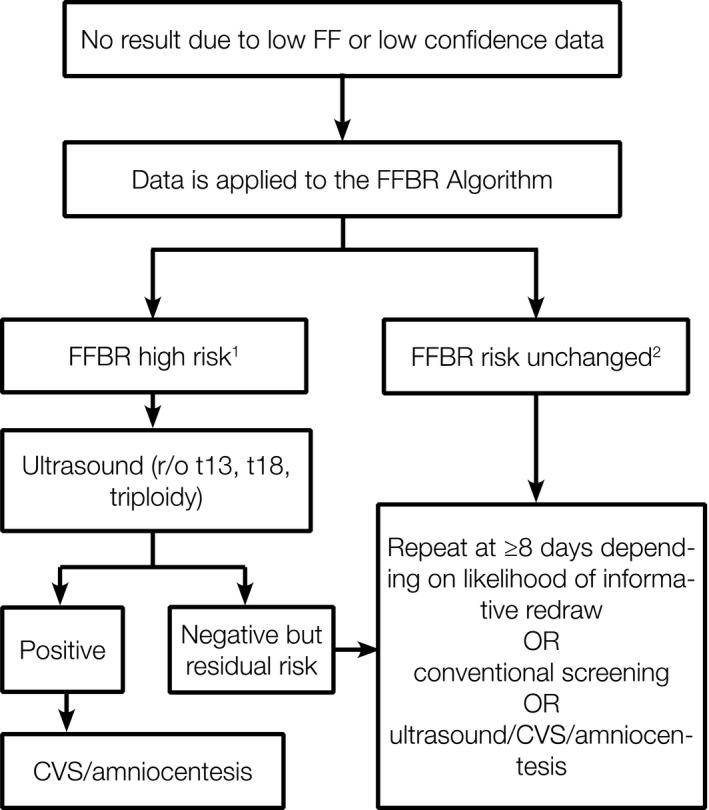
Use of the fetal fraction‐based risk (FFBR) and likelihood of informative redraw algorithms for women without prior ultrasound or conventional screening results. 1. FFBR high risk ≥ 1 in 100 for t13, t18, or triploidy. Reported as: ‘High risk due to fetal DNA fraction with a risk score of 1 in 17 for trisomy 18, trisomy 13, or triploidy’. 2. FFBR risk unchanged. Reported as: ‘No results, a repeat specimen may be considered’. Abbreviations: CVS, chorionic villus sampling; FFBR, fetal fraction‐based risk; r/o. rule out; t13, trisomy 13; t18, trisomy18

Fetal fraction‐based risk high‐risk reports state that women have a risk score of 1 in 17 for trisomy 18, trisomy 13, or triploidy which is based on the positive predictive value for the test (McKanna et al., 2019). These women should therefore be counseled that their risk is significantly increased and early detection is advantageous. Most affected pregnancies should be identifiable by ultrasound, particularly when performed in the second trimester. Ultrasound information is important in helping patients to decide whether they would want diagnostic testing. The FFBR algorithm does not provide information about risk for trisomy 21 and monosomy X. To address this risk, women with high FFBR and normal ultrasound could consider conventional prenatal screening or a repeat NIPT assuming that there are no additional risk factors, and a comprehensive ultrasound evaluation is normal. An estimate of redraw success for this group of women, after exclusion of cases with abnormal ultrasound findings, is not currently available. However, a previously published estimate based on all cases with uninformative draws could be used (Benn et al., 2018), recognizing that affected pregnancies are a minor proportion of all uninformative cases. Figure 2 summarizes how we suggest FFBR and probability of informative redraw algorithms are used.

### 4.3 Limitations

The dataset used in this analysis was based on a relatively high‐risk group of women with a total of 54/1673 (3.2%) suspected chromosome abnormalities in the cases with an informative redraw (Table 3). The proportion of cases that would classified as high FFBR may be less in a cohort of women with lower a priori risks. The actual pregnancy outcomes were unknown for the cases included in this study. However, the FFBR algorithm was developed from an initial training set of samples and then validated based on a robust set of cases with uninformative results due to low FF (McKanna et al., 2019). The inclusion of chromosomally abnormal cases in the regression analysis was appropriate because the purpose of redrawing was to detect abnormality. The predictive model for informative redraws has not been prospectively validated.

The patient management pathways in Figure 2 do not consider all the clinical scenarios that may influence the decision to pursue a primary NIPT, a redraw, or consideration of invasive diagnostic testing. For example, deciding on a redraw may also be based on the patient's age‐related risk, gestational age (and the available time for a follow‐up diagnostic procedure), risks based on ultrasound or prior conventional screening, the overall health of a patient, medication use, pregnancy history, and family history. Some of these factors may also increase or decrease risk for a chromosome abnormality prior to NIPT and are not taken into consideration in NIPT risk assessments. However, all these factors are important for counseling a patient about the best next step for them: redraw NIPT, other screening, invasive diagnostics, or no further testing. All patients have the ongoing choice to determine the type of information that is important to them regarding their pregnancy and the health of their fetus. We recognize that the inability to provide a NIPT result can be stressful and frustrating for patients and even though risk may be unchanged, some women will opt to receive additional ultrasound examinations or invasive tests that would not have otherwise been performed. These detract from the overall (effective) specificity of the screening process. An additional current limitation of the FFBR algorithm is that it does not provide separate risks for trisomy 13 and trisomy 18.

### 4.4 Research recommendations

FF represents the balance between trophoblastic and maternal cell apoptosis or necrosis. Maternal factors that have been associated with altered FF include intrahepatic cholestasis, (Vlkova et al., 2016) autoimmune disease (Chan et al., 2014; Hui, Tan, Tan, & Tan, 2016), use of medications such as heparin (Burns et al., 2017), and pregnancy complications (Dugoff et al., 2016; Krishna, Badell, Loucks, Lindsay, & Samuel, 2016; Levine et al., 2004). Placental disorders unrelated to chromosome abnormality, notably those leading to fetal growth restriction, might be associated with uncharacteristically low FF. As these associations become better understood, it is possible that FF can be used as a biomarker to help identify pregnancies at increased risk for these conditions, in addition to chromosome abnormalities. These future studies should also allow refinement of the predictive model for an informative NIPT redraw.

### 4.5 Overview

In summary, the FFBR algorithm and predictive model for redraw success provide complimentary information to assist providers in counseling women whose initial Natera SNP‐based NIPT does not return a result. Those women with high FFBR should be offered genetic counseling, ultrasound, and the option of diagnostic testing prior to consideration of a repeat NIPT, while risk‐unchanged women can consider redraw based on their personal likelihood for an informative result. This management combination substantially decreases the number of women with uninformative results.

## COMPLIANCE WITH ETHICAL STANDARDS

### Conflicts of interest

Peter Benn and Kimberly Martin are paid consultants and hold stock options in Natera, Inc. Trudy McKanna, Elizabeth Valenti, Paul Billings, and Zachary Demko are employees and own stock in Natera, Inc. The work was funded by Natera, Inc.

### Human studies and informed consent

The study was considered ‘exempt’ by an investigational review board (Ethical and Independent Review Services, Study Number: 17113‐01).

### Animal studies

No animal studies were carried out.

## AUTHOR CONTRIBUTIONS

Peter Benn was involved in the design, analysis, and writing of the manuscript. Kimberly Martin, Trudy McKanna, and Elizabeth Valenti provided clinical and counseling perspectives. Paul Billings was involved with funding the project. Zachary Demko has been involved in all aspects. All authors approved the final version of the manuscript and vouch for the accuracy and integrity of the content.
