# Supplementary material for: Combining the use of a fetal fraction‐based risk algorithm and probability of an informative redraw in noninvasive prenatal testing for fetal aneuploidy
Source: J Genet Couns. 2019 Dec 23;29(5):800–6. doi: 10.1002/jgc4.1208 (PMC7586960; doi:10.1002/jgc4.1208)
Supplement: Supplementary file 1 [file JGC4-29-800-s001.docx]

**Supplemental Table 1**. Binary logistic regression analysis output data for women with unchanged risk by the algorithm.

|  | | | | | | | | |
| --- | --- | --- | --- | --- | --- | --- | --- | --- |
|  | Regression coefficient (a) | S.E. | Wald | df | Sig. | Odds ratios (b) | 95% C.I.for odds ratios | |
|  |  |  |  |  |  |  | Lower | Upper |
| First draw fetal fraction (FF) (%)  Maternal weight (MW) (lbs)  Time between draws (t) (days)  Gestational age (days)  Constant | 0.909 | 0.086 | 111.220 | 1 | <0.001 | 2.481 | 2.095 | 2.937 |
|  | -0.005 | 0.001 | 13.885 | 1 | <0.001 | 0.995 | 0.992 | 0.998 |
|  | 0.059 | 0.011 | 31.607 | 1 | <0.001 | 1.061 | 1.039 | 1.083 |
|  | 0.002 | 0.004 | 0.213 | 1 | 0.644 | 1.002 | 0.994 | 1.009 |
|  | -2.065 | 0.532 | 15.066 | 1 | <0.001 | 0.127 |  |  |

The logistic regression model had a -2 Log likelihood ratio = 1608, Cox and Snell R Square = 0.197 and Nagelkerke R Square = 0.271.

(a)  The regression coefficients are used to calculate the probability of an informative redraw for any continuous scale combination of FF, MW, and t. The probability of an informative redraw is given by the formula:
Informative probability = 1/(1+EXP(-(-2.065+(0.909*FF) +(0.005*MW)+(0.909*t))))

Example data for the probabilities are shown in Table 4.

(b)  The Odds Ratios indicate the change in the informative redraw rate per unit change in a variable. For example, a 1% increase in FF will increase the chance of an informative redraw by 2.481-fold (95% CI).

Abbreviations: S.E., standard error; Wald, Wald statistic; df, degrees of freedom; Sig, significance; C.I., confidence intervals.

**Supplemental Figure 1.** Flow chart showing the study cohort.

Low Risk by SNP Analysis

N=664

High Risk by SNP Analysis

N=34

(16 t21

6 t18

5 t13

3 SCA

4 multiple GA or triploidy)

Informative Redraw

N=698

Low Risk by SNP Analysis

N=955

High Risk by SNP Analysis

N=20

(10 t21

1 t18

2 t13

7 SCA)

Uninformative Redraw

N=449

Informative Redraw

N=975

Uninformative

Redraw

N=522

FFBR High Risk Score

N=1,147

Incomplete information

N=315

Initial Sample, No Result with Second Sample Received

N=2,959

Calculate FFBR & likelihood of informative redraw

N= 2,644

FFBR Risk Unchanged Score

N=1,497

Abbreviations: FFBR, fetal fraction-based risk; SNP, single nucleotide polymorphism; t21, trisomy 21; t18, trisomy 18; t13, trisomy 13; SCA, sex chromosome abnormality; GA; gestations.
